# Antioxidant and Anti-Obesity Activities of *Polygonum cuspidatum* Extract through Alleviation of Lipid Accumulation on 3T3-L1 Adipocytes

**DOI:** 10.4014/jmb.1910.10040

**Published:** 2019-12-13

**Authors:** Da-Hye Choi, Joon-Hee Han, Keun-Hyung Yu, Min Hong, Sun-Yeop Lee, Ka-Hee Park, Soo-Ung Lee, Tae-Hyung Kwon

**Affiliations:** Department of Research and Development, Chuncheon Bio-industry Foundation (CBF), Chuncheon 24232, Republic of Korea

**Keywords:** 3T3L1 cells, lipogenesis, adipogenesis, antioxidant, *Polygonum cuspidatum*

## Abstract

Natural products are widely used due to their various biological activities which include antiinflammatory, antioxidant, and anti-obesity effects. In this study, we determined the antioxidative and anti-obesity effects of *Polygonum cuspidatum* 50% ethanol extract (PEE). The antioxidative effect of PEE was evaluated using its radical scavenging activity, total phenolic content, and reducing power. The anti-obesity effect of PEE was investigated using 3T3-L1 adipocytes. The antioxidative activity of PEE was progressively increased in various concentrations, mainly due to the presence of phenolic compounds. PEE also alleviated lipid accumulation on 3T3-L1 adipocytes and downregulated the mRNA and protein production of adipogenesis-related (SREBP-1c, PPARγ, C/EBPα) and lipogenesis-related (aP2, FAS, ACC) markers. Furthermore, we found that the inhibitory effect on lipid accumulation via PEE was caused by the alleviation of NF-κB, p38 MAPK, ERK1/2, and JNK at the protein level. Taken together, our results imply that PEE is a potential antioxidant that can prevent obesityassociated disorders.

## Introduction

Obesity is the excessive or abnormal accumulation of fat, which can lead to dyslipidemia, hyperlipidemia, diabetes, cardiovascular disease, cancer, liver disease, hypertension, and psychological disorders [1, 2]. Recently, obesity has been associated with an increase in metabolic syndrome prevalence [3-5]. Metabolic syndrome increases the risk of diseases such as brain stroke, type 2 diabetes, and cardiovascular disease [4]. These conditions include increased blood glucose levels, high blood pressure, excess body fat, and abnormal cholesterol or triglyceride (TG) levels. TGs are the major component of lipids in humans. The excess storage of TGs in adipocytes can cause obesity; moreover, increased TG levels in the blood, liver, and muscle tissues of overweight individuals can cause pathological disorders [6-8].

Adipocytes, also known as fat cells, are composed of adipose tissue (AT) and play a leading part in the inhibition of lipid homeostasis and metabolism. The increase in AT mass is caused by an increase in adipocyte numbers (hyperplasia) and size (hypertrophy). The mouse embryonic fibroblast 3T3-L1 preadipocytes that can differentiate into mature adipocytes following exposure to adipogenic inducers such as insulin, IBMX, and DEX [9, 10]. These inducers activate complex signaling pathways and various transcription factors. Natural products can enhance anti-obesity effects by downregulating the expression of adipogenic transcriptional factors such as SREBP-1c, C/EBPα, and PPARγ in 3T3-L1 preadipocytes [11-13]. ACC, FAS, and FABP4 (also known as aP2) are upregulated during lipogenesis [14, 15], whereas AMPK, a central regulator of energy homeostasis, can suppress glucose and lipid synthesis by reducing the production of transcription factors and metabolic enzymes such as FAS and ACC [16]. In addition, various MAPK signaling pathways such as ERK1/2, JNK, and p38 MAPK have been associated with obesity [17]. NF-κB plays an important role in inflammatory reaction and also might be associated with obesity-related pathology [18].

Various herbal extracts and phytochemicals have been used to inhibit adipogenesis and induce apoptosis in mature adipocytes [19, 20]. *Polygonum cuspidatum* (also known as *Reynoutria japonica*) is a herbaceous, perennial plant species of the family Polygonaceae and is found in China, Japan, and Korea. The plant root is widely used to treat menoxenia, skin burn, gallstone, hepatitis, inflammation, osteomyelitis, and obesity [21-23]. The main phenolic compounds found in the roots of *P. cuspidatum* related to these properties are emodin, resveratrol, and piceid [24]. However, few studies have investigated the signaling pathways modulated by *P. cuspidatum* to affect the proliferation and differentiation of adipocytes and whether these pathways are associated with transcription factor-dependent or independent mechanisms, as well as MAPK.

In the present study, we evaluated the antioxidative activity of *P. cuspidatum* 50% ethanol extract (PEE) using DPPH and ABTS methods, as well as its anti-obesity effect using 3T3-L1 adipocytes, by measuring the lipid accumulation and determining the mRNA expression of adipogenesis (*C/EBP*α, *PPAR*γ, and *SREBP-1c*) and lipogenesis (*FAS*, *ACC*, and *aP2*) markers. In addition, protein production of these markers and the phosphorylation state of MAPKs (P38, Erk1/2, and JNK) and NF-κB were determined.

## Materials and Methods

### Preparation of Sample and Chemicals

*P. cuspidatum* was obtained from Gwang-Myoung (Korea) in April 2019. The *P. cuspidatum* root sample was pulverized to an 80 mesh size using the DA280-S grinder (Daesung Artlon, Korea), and freeze dried (Ilshin, Korea) to obtain powder. ABTS was obtained from Wako Industries, Ltd. (Japan). Folin-Ciocalteu reagent, potassium ferricyanide, trichloroacetic acid, ferric chloride, butylated hydroxyanisole, ascorbic acid, EDTA, IBMX, glycerol, MTT, dimethyl sulfoxide, dexamethasone, emodin, piceid, and resveratrol were obtained from Sigma Chemical Co., LLC. FBS and DMEM were obtained from Gibco Laboratory (USA). Antibodies against PPARγ (#2435), C/EBPα (#8178), FAS (#3180), ACC (#3676), NF-kB (Phospho-p38 (#9212), p38 (#9211), Phospho-ERK (#4370), ERK (#9102), Phospho-JNK (#9251), JNK (#9252), and β-actin (#4970), as well as anti-rabbit IgG (#7074) and anti-mouse IgG (#7076), were purchased from Cell Signaling Technology (USA). An anti-SREBP1-c (ab28481) antibody was obtained from Abcam Inc. (USA).

### Determination of Total Polyphenols 

The TPC of PEE was measured according to the Folin-Denis method [25]. Briefly, Folin-Denis phenol reagent (50 μl) was added to 50 μl PEE. It was then incubated for 3 min. Thereafter, 10% Na_2_CO_3_ (50 μl) was added and further incubated for 60 min. This solution, was then quantified at 760 nm using a SpectraMax M5 plate reader (Molecular Devices, USA). The TPC of PEE is shown as mg gallic acid equivalents (GAE)/g. Each sample assay was performed in triplicate.

### Antioxidant Activity Assays-DPPH

The DPPH radical scavenging assay was performed using the Blois method [26]. Briefly, 50 μl of PEE was added to 100 μl of 0.2 mM DPPH solutions, vortexed, and left to stand at room temperature for 10 min. The solution was quantified at 517 nm. Each sample assay was performed in triplicate. The results were compared with BHA and AA and the activity was calculated by the following formula:

$$\mathrm{scavenging}\mathrm{effect}(\%)=(1-\frac{A_{S}-A_{\mathrm{SB}}}{A_{C}})\times 100.$$

### Antioxidant Activity Assays-ABTS

The ABTS radical cation decolorization was performed by Re *et al*. [27]. Briefly, 7 mM of ABTS was mixed with 2.45 mM and left to at room temperature in dark condition for 24 h. Fifty microliters of PEE was added to 100 μl of ABTS solution, vortexed, and left to stand at room temperature for 10 min. The solution was quantified at 734 nm. Each sample assay was performed in triplicate. The results were compared with BHA and AA and the activity was calculated by the following formula:

$$\mathrm{scavenging}\mathrm{effect}(\%)=(1-\frac{A_{S}-A_{\mathrm{SB}}}{A_{C}})\times 100.$$

### Antioxidant Activity Assays-Reducing Power

The reducing power of PEE was determined according to the Oyaizu method [28]. A reaction mixture containing 1 ml of PPE and 1 ml of 1% potassium ferricyanide was incubated in a water bath at 50°C for 20 min. After incubation, 10% trichloroacetic acid was added, centrifuged at 3,000 ×g for 10 min. Next, 1 ml of upper layer was added to 1 ml distilled water and 0.1 ml of 0.1% ferric chloride. The solution was quantified at 700 nm. Each sample assay was performed in triplicate. The results were compared with BHA and AA.

### Cell Culture for Adipocyte Differentiation

3T3-L1 preadipocytes were grown in T-flask with DMEM containing 10% neuborn calf serum (FBS) at 37°C in 5% CO_2_ atmosphere. Then, cells were subcultured after being grown to more than 80-90% confluence. Two days after confluence (day 0), cells were induced with an MDI mixture of 0.5 mM IBMX, 10 μg/ml insulin and 1 μM DEX in DMEM containing 10% FBS for 2 days (day 2). Cells were then maintained in DMEM supplemented with 10% FBS and 1 μg/ml insulin for another 2 days (day 4), followed by culturing with DMEM with 10% FBS for an additional 4 days (day 8). Test samples were added in medium at various concentrations throughout the whole culture period (day 0-8).

### Cell Counting Kit-8 (CCK-8) Assay for Cell Viability

The cell viability of samples was measured using Cell Counting Kit-8 (Dojindo, Japan). Briefly, the cells were seeded at 10^4^cells/well onto flat bottomed 96-well plates and treated with test samples at various concentrations. After incubation for 48 h, the cells were added to CCK-8 solution (10 μl/well) in each well and incubated at 37°C for 4 h. After incubation, the 96 well-plate was measured at 450 nm.

### Oil Red O Staining

Cells were washed three times with phosphate buffered saline (PBS) and fixed with 4% formaldehyde for 30 min. The fixed cells were washed gently with distilled water. Oil Red O (0.5% in 60%isopropanol) was diluted with water (3:2), filtered through Whatman paper, and incubated with the fixed cells for 1 h at room temperature. Plates were rinsed three times with distilled water and the stained fat droplets in the adipocytes were visualized by inverted microscopy and photographed. Spectrophotometric analysis of the stain was performed by dissolving the stained lipid droplets with isopropanol and measuring at A_520_ nm.

### Western Blot Analysis

The cells were harvested using a cell scraper (SPL Co., Korea) and then washed twice in PBS and extracted using PRO-PREP^TM^ (iNtRON Biotechnology, Korea). The protein concentration measurements were performed according to the method of the Pierce BCA Protein Assay (Thermo Fisher Scientific, USA). Protein was separated using an SDS-PAGE gel and transferred to PVDF membranes. The membranes were immersed in TBS-T buffer (0.5 M tris-HCl of pH 7.5, 1.5 M NaCl and 0.1% tween 20) containing 3% fat milk for 1 h to nonspecific for blocking. Primary antibodies were probed in membranes for 16 h with PPARγ, C/EBPα, SREBP-1c, FAS, ACC, NF-κB, β-actin and MAPK (p38, Erk1/2, JNK, AMPKα with phosphatase). After washing with TBS-T buffer, the membranes were incubated with secondary antibody for 1 h. Immunostaining of antibodies was detected using the Immobilon (Millipore Corporation, USA) and imaged with an LAS-4000 (Japan). The monoclonal rabbit β-actin was used as a comparative control.

### Real-Time Polymerase Chain Reaction (RT-PCR)

The RNA was extracted using the RNA Isolation Kit (Roche Molecular Systems, Inc., USA) following the protocol supplied with the product. The purity of RNA was calculated with the ratios of absorbance at 260 and 280 nm using a spectrophotometer (Biochrom Libra S32, UK). The 1 μg of RNA was reverse transcribed into cDNA using the cDNA Synthesis Kit (Roche Molecular Systems, Inc.). The oligonucleotide primers are shown in Table 2 [29]. Total reaction volumes were set up in 20 μl with 1 μl of each primer, 10 μl SYBR Mixture (Roche Molecular Systems, Inc.), 2 μl template DNA, and 6 μl RNase-free water. The cycling conditions were as follows: 5 min at 95°C, 45 cycles of 15 sec at 95°C, 15 sec at 52°C, 30 sec at 72°C. The analysis of PCR products was performed using the LightCycler 480 Instrument II (Roche Molecular Systems, Inc.). For relative quantitative analysis, the data were normalized using β-actin and phosphatase genes, and then the fold-changes of gene expression were calculated based on comparison with the reference gene.

### Analysis of PEE Using High-Performance Liquid Chromatography (HPLC)

The analysis of major compounds in PEE was conducted using HPLC (Shimadzu, Japan). The YMC gel ODS A302 column (YMC Separation Tech Ltd., 4.6 × 250 mm) was used for the analysis of piceid, resveratrol, and emodin. The mobile phases were 0.1%formic acid (v/v) in water (solvent A) and 100% acetonitrile (solvent B). All reagents used were HPLC grade. The gradient followed the given order as follows: 5 min 8%→15% B, 5–10 min 15%→30% B, 10–20 min 30%→70, and 20–30 min 70%→100% B. The separated compounds were detected with a UV detector at 280 nm.

### Statistical Analysis

In this study, all statistical analyses used were performed using SPSS 18.0 statistical software (SPSS, USA). Statistical significance was determined by one-way analysis of variance followed by Duncan’s multiple range test for multiple comparisons. Statistically significant differences were considered at *p* < 0.05. All experimental data are presented as the mean ± standard deviation (SD).

## Results

### Antioxidative Activities of PEE

PEE was evaluated by measuring total phenolic content, antioxidative activities using DPPH and ABTS radical scavenging activity, and reducing power. The total phenolic content of PEE was 92.39 mg GAE/g (Table 1). Table 1 shows the DPPH and ABTS radical scavenging activity and reducing power of PEE. Both DPPH and ABTS radical scavenging activity and reducing power were increased in a concentration-dependent manner. Compared to that at 100 μg/ml, the value for DPPH was 46.14%, whereas those for BHA and ascorbic acid were 77.81% and 91.29%, respectively. In terms of reducing power, the absorbance values of PEE, BHA, and ascorbic acid were 0.121, 0.486, and 0.532, respectively, at the same concentration.

### Inhibitory Effect of PEE on Adipocyte Differentiation

**Fig. 1:**
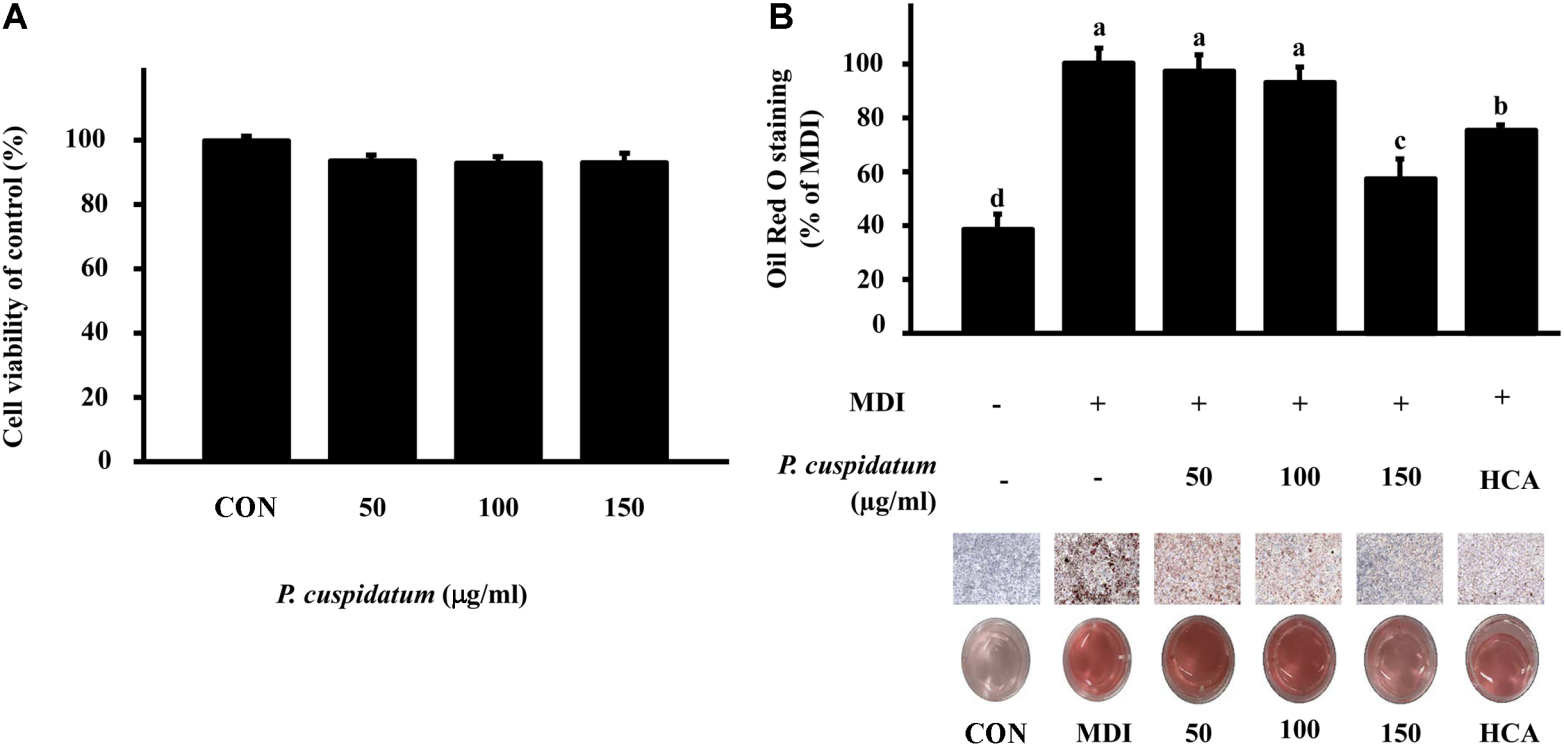
Cell viability and Oil Red O staining of 3T3-L1 adipocytes of PEE. (**A**) Cell viability in 3T3-L1 adipocytes of PEE. Cell viability was measured using MTT assay. (**B**) Effect of PEE on lipid accumulation in 3T3-L1 adipocytes. Differentiation of 3T3-L1 preadipocytes was stained with Oil Red O and photographed (upper panel: 200× magnification, lower panel: 3T3-L1 cell lipid drop differentiated in 24-well plate). Data represent the means ± SD in triplicate. MDI: DMEM with 3-isobutyl-1-methylxanthine, dexamethasone, and insulin. ^a-d^: Means the different letters on the bars are significantly different at *p* < 0.05.

To evaluate the inhibitory activity of PEE towards 3T3-L1 adipocyte differentiation, we initially measured cell viability at several concentrations of PEE using the MTT assay. Our results showed that the viability of 3T3-L1 cells was not decreased upon treatment with 50, 100, and 150 μg/ml of PEE (Fig. 1A). Next, we treated 3T3-L1 adipocytes with varying concentrations of PEE and stained them with Oil Red O. As shown in Fig. 1B, PEE significantly reduced lipid accumulation during adipocyte differentiation in a concentration-dependent manner. Moreover, lipid accumulation in the cells treated with 150 μg/ml PEE was decreased by more than 43% compared to that in control cells (treated with MDI only).

### Effects of PEE on mRNA Expression and Protein Production of Adipogenesis-Related Markers

**Fig. 2:**
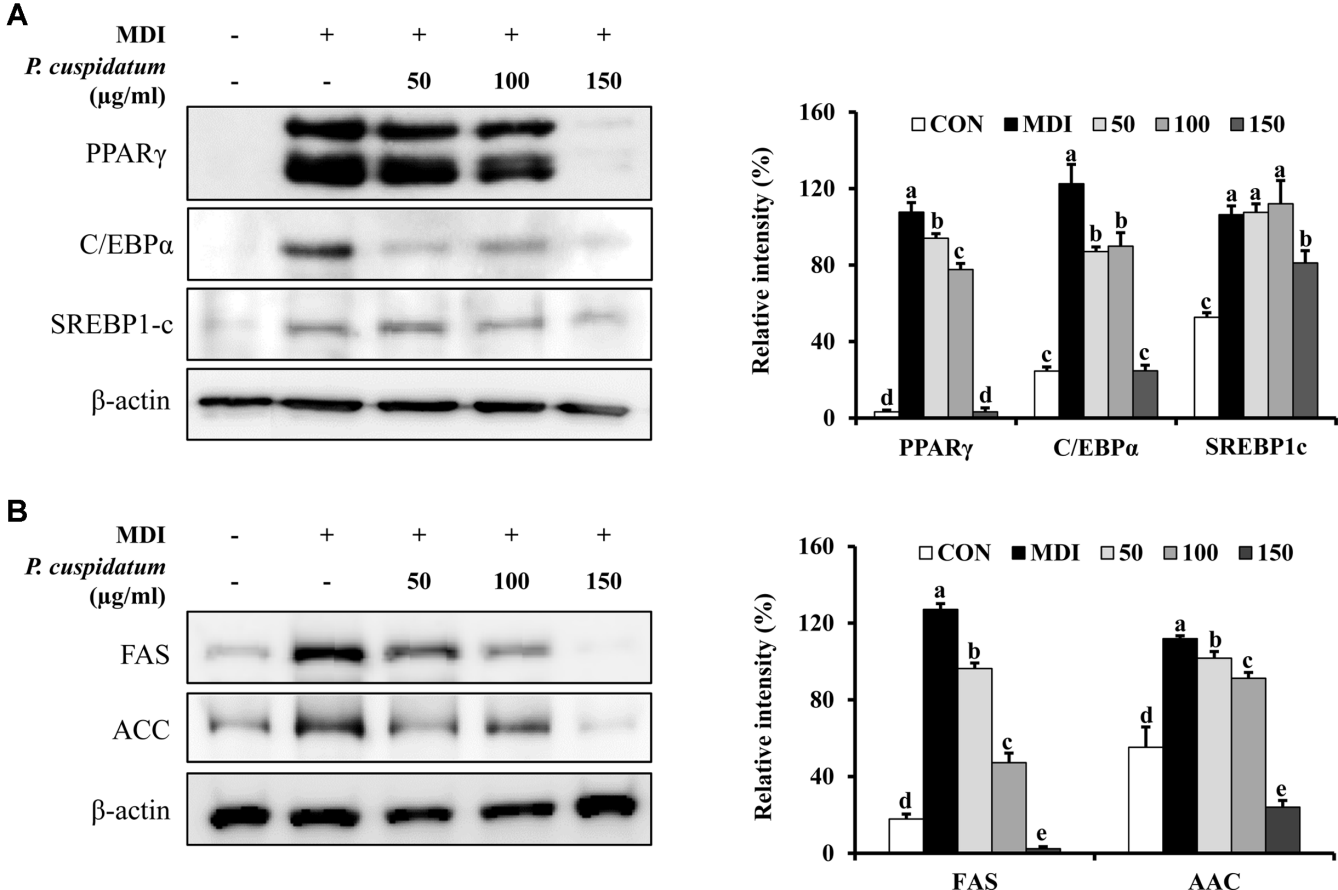
Inhibitory effects of PEE on the protein production level of specific transcription factors in 3T3-L1 preadipocytes after 8-day differentiation. The protein production levels as values relative to β-actin of (**A**) adipogenesis (PPARγ, C/EBPα, and SREBP-1c) and (**B**) lipogenesis (FAS and ACC) transcription factors were measured using western blot analysis. Each value is expressed as the mean ± SD. CON: undifferentiated preadipocyte; MDI: differentiated adipocyte; 50: adipocyte was treated with 50 μg/ml of PEE; 100: adipocyte was treated with 100 μg/ml of PEE; 150: adipocyte was treated with 150 μg/ml of PEE; PPAR γ, peroxisome proliferator-activated receptor gamma; C/EBPα, CCAAT/enhancerbinding protein alpha; SREBP-1c, sterol regulatory element binding protein-1c; FAS, fatty acid synthase; ACC, acetyl-CoA carboxylase. Data represent the means ± SD in triplicate. ^a-e^: Means the different letters on the bars are significantly different at *p* < 0.05.

**Fig. 3:**
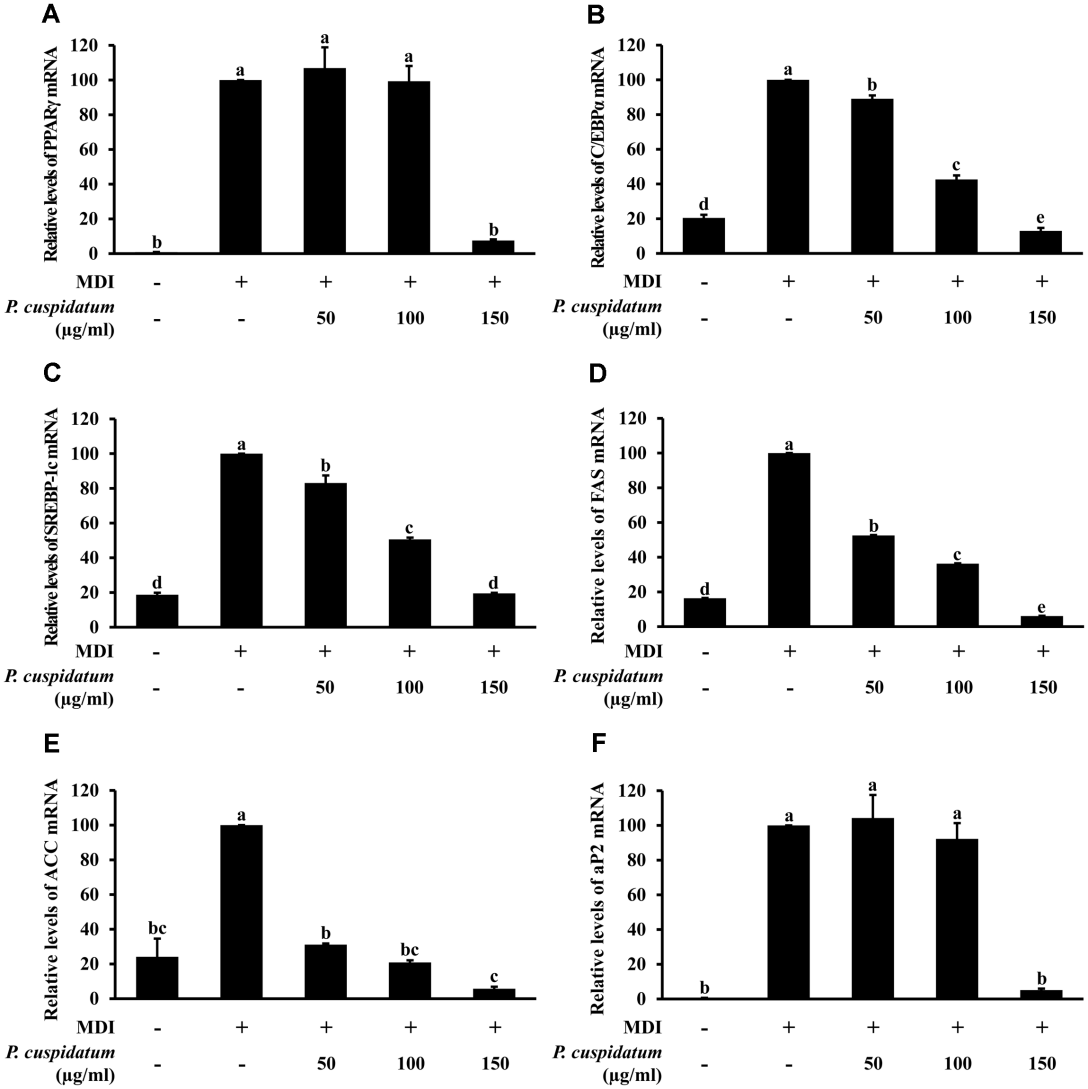
Inhibitory effects of PEE on mRNA expression levels of (**A**) PPARγ, (**B**) C/EBPα, (**C**) SREBP-1c, (**D**) FAS, (**E**) ACC, and (**F**) aP2 in 3T3-L1 preadipocytes after 8-day differentiation. MDI: differentiated adipocyte; PPAR γ, peroxisome proliferator-activated receptor gamma; C/EBPα, CCAAT/enhancer-binding protein alpha; SREBP-1c, sterol regulatory element binding protein-1c; FAS, fatty acid synthase; ACC, acetyl-CoA carboxylase; aP2, adipocyte protein 2. Data represent the means ± SD in triplicate. ^a-e^: Means the different letters on the bars are significantly different at *p* < 0.05.

The expression of adipogenesis-related genes in fully-differentiated adipocytes was analyzed using real-time PCR and western blotting. As shown in Fig. 2A and Figs. 3A–3C andhe expression of PPARγ, C/EBPα, SREBP-1c, and aP2 was significantly increased in 3T3-L1 cells treated only with MDI; however, the mRNA expression and protein production of these markers were significantly decreased when cells were treated with 150 μg/ml PEE during the differentiation-inducing process.

### Effects of PEE on mRNA Expression and Protein Production of Lipogenesis-Related Markers 

As shown in Fig. 2B and Figs. 3D–3F, PEE (150 μg/ml) significantly inhibited the mRNA expression and protein production of FAS, ACC, and aP2. These results were similar to those obtained for adipogenesis-related genes and lipid accumulation based on Oil Red O staining.

### Effects of PEE on NF-kβ, ERK, JNK, and p38 Protein Production

**Fig. 4:**
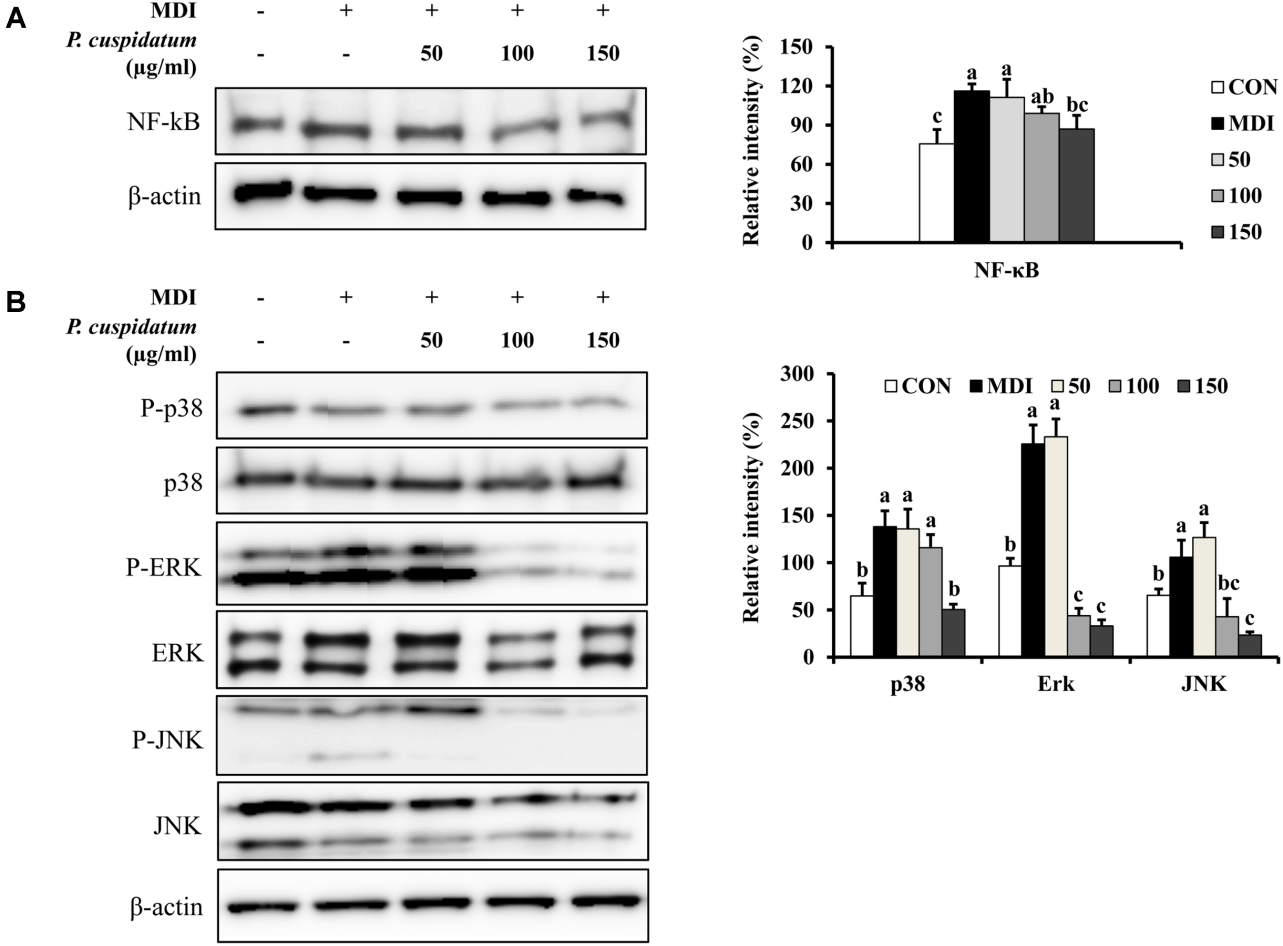
Effects of PEE on the protein production levels for NF-kB and MAPK signaling pathway in 3T3-L1 preadipocytes after 8- day differentiation. (**A**) The protein production level as values relative to β-actin of NF-kB was measured using western blot analysis. (**B**) p38, ERK, JNK and their phosphorylated forms were assessed by the western blot analysis. Each value is expressed as the mean ± SD. CON: undifferentiated preadipocyte; MDI: differentiated adipocyte; 50: adipocyte was treated with 50 μg/ml of PEE; 100: adipocyte was treated with 100 μg/ml of PEE; 150: adipocyte was treated with 150 μg/ml of PEE; NF-kβ, nuclear factor kappa-light-chain-enhancer of activated B cells; p38, p38 mitogen-activated protein kinase; ERK, extracellular signal-regulated protein kinases 1 and 2; JNK, c-Jun N-terminal kinase. Data represent the means ± SD in triplicate. ^a-c^: Means the different letters on the bars are significantly different at *p* < 0.05.

To confirm the anti-obesity activity of PEE, we investigated its effect on the protein production of NF-κB, p38, ERK, and JNK. In 3T3-L1 adipocytes, NF-κB levels were significantly reduced only at 150 mg/l compared to those in the control group (Fig. 4A). This result suggests that PEE affects the adipogenic process in adipocytes by downregulating NF-κB expression. PEE also markedly decreased the MDI-induced phosphorylation of p38, ERK, and JNK (Fig. 4B).

### Analysis of PEE Composition Using HPLC

**Fig. 5:**
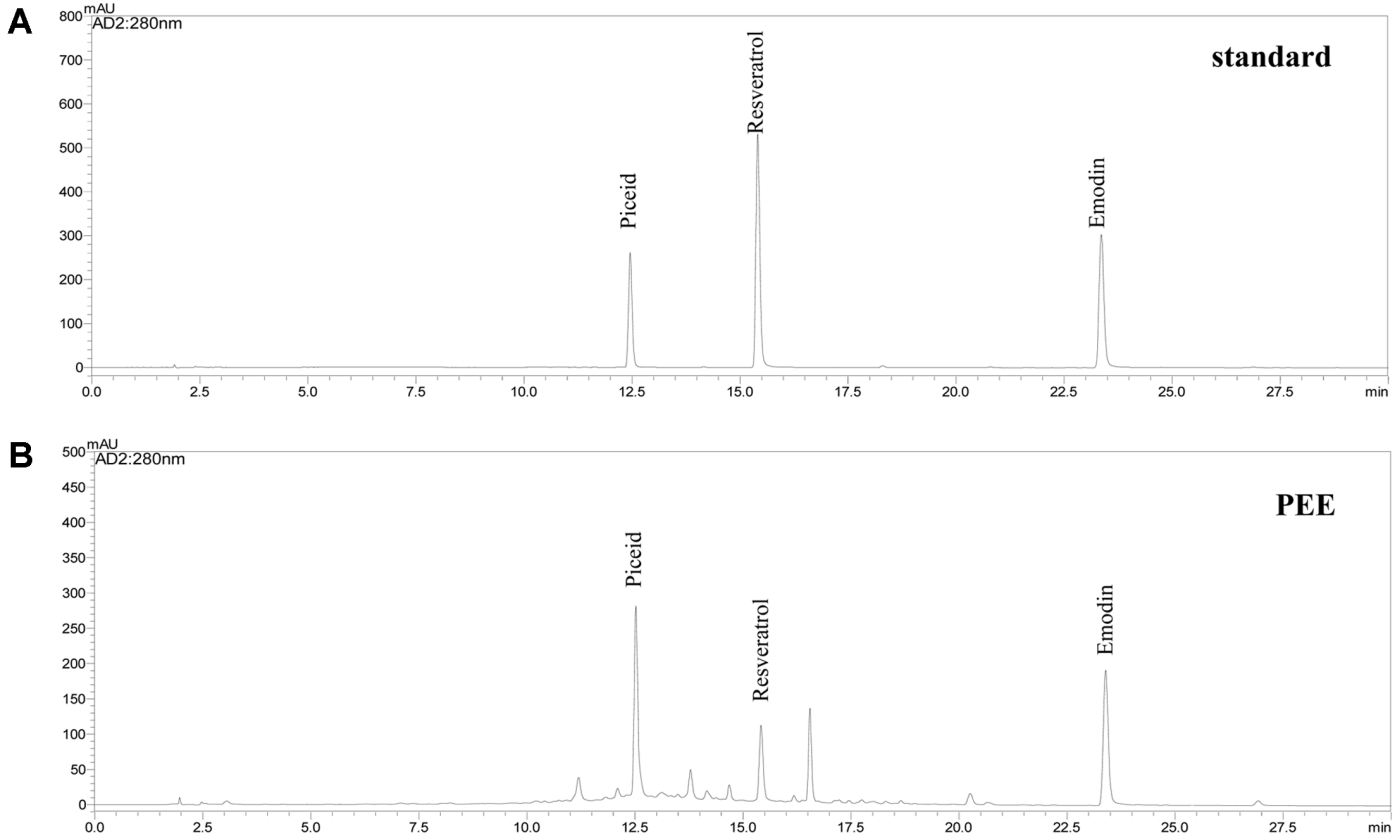
HPLC traces of piceid, resveratrol, and emodin in PEE.

We performed HPLC to determine the content of the major compounds in PEE. Fig. 5 shows the profile of major compounds in PEE. As a result, HPLC analysis revealed the presence of emodin, piceid, and resveratrol in PEE, and the values were 27.8, 41.2, and 8.3 mg/g, respectively (data not shown).

## Discussion

ROS can generate activated oxygen such as hydrogen peroxide (H_2_O_2_), singlet oxygen (^1^O_2_), hydroxyl radical (^.^OH), and superoxide anion (O_2_^.−^), and it can be implicated in numerous diseases [30]. Excessive ROS can cause DNA damage, enzyme inactivation, cellular necrosis and apoptosis, and lipid peroxidation, which in turn, can lead to inflammation, cancer, cardiovascular diseases, neural diseases, aging, arteriosclerosis, and rheumatism [31]. Antioxidants are important molecules that provide protection against the deleterious effects of ROS, and are therefore frequently used in the medicinal and food industries. The interest in identifying natural antioxidants from plants, seaweed, microorganisms, and other materials has been increasing recently [30-32].

Recent studies have demonstrated that secondary metabolites such as polyphenols, terpenes, and carotenoids possess antioxidative, anti-inflammatory, and anti-obesity activities [33, 34]. Several studies have also reported the antioxidative activity of saponin, pro-anthocyanin, and alkaloid [35, 36].

Obesity is caused by nutritional imbalances; in other words, an excessive intake of calories and lack of energy expenditure can result in obesity. Moreover, obesity can cause hypertension, type II diabetes, cancer, fatty liver disease, and cardiovascular diseases [37]. Oil Red O is used to stain neutral lipids and adipocytes and visualize fat droplets [38]. Here, anti-obesity activity of PEE was assessed using Oil Red O staining on 3T3-L1 adipocytes. Adipocyte differentiation was investigated using MDI mixtures with PEE (50, 100, and 150 μg/ml) for 8 days. Our results imply that PEE alleviated lipid accumulation in the differentiated 3T3-L1 adipocytes.

From the aforementioned results, we concluded that PEE can effectively regulate adipocyte differentiation on 3T3-L1 cells without toxicity. Therefore, we next studied whether PEE affects the activation of adipogenic and lipogenic transcription factors. Adipocyte differentiation is a three-stage process and is regulated via transcription factors at each stage [39, 40]. First, C/EBPβ and C/EBPδ are recruited by the differentiation inducers IBMX and DEX, respectively. CEBPα and PPARγ are expressed during the mid-differentiation stage; and as adipogenesis transcription factors they were downregulated by PEE. Next, we further studied whether PEE inhibits the expression of lipogenic enzyme. FAS, ACC, and aP2 are expressed during lipogenesis or late-stage adipocyte differentiation and are overexpressed only in mature adipocytes. In our results, PEE was shown to downregulate mRNA expression and protein production.

The NF-κB family plays an important role in inflammation and the reaction to obesity [18]. Moreover, the MAPK pathways play an essential role in regulating transcription factor expression [17]. MAPK consists of three pathways, namely ERK, JNK, and p38. The MAPKs are a group of protein kinases that regulate cellular growth, differentiation, stress responses, and cancer progression [41]. A recent study reported that the MAPK pathways can regulate all stages of adipogenesis in stem cells and adipocytes [42]. Further, the reduced activation of ERK, JNK, and p38 in pre-adipocytes showed alleviation of adipocyte differentiation. Furthermore, the MAPK signaling pathway regulates the mRNA expression of PPARγ and C/EBPα during adipogenesis in 3T3-L1 cells [43]. Therefore, our result suggested that PEE alleviates adipogenesis and lipogensis in adipocyte differentiation cells through inhibition of ERK, JNK, and p38 phosphorylation.

In the present study, we also performed HPLC to identify the main components of PEE. Emodin is an anthraquinone of aromatic organic compounds, including aloe, senna, cascara, and rhubarb. In addition, this has a variety of pharmacological effects, including laxative, anti-allergic, anti-inflammatory, anti-cancer, and anti-diabetic. Emodin affects 3T3-L1 cell differentiation in a concentration-dependent manner and inhibits the activity of FAS and PPARγ agonists [44, 45]. A recent study showed that emodin can regulate glucose homeostasis in vivo by activating AMPK [46]. Taken together, emodin is a potential drug candidate for the treatment of type II diabetes and other obesity-related metabolic diseases. Moreover, Kuo *et al*.[24] reported the phenolic compounds such as emodin, piceid, and resveratrol in *P. cuspidatum*, as well as optimized ultrasound-assisted extraction. These results were similar to our data.

In conclusion, PEE exerts an antioxidative effect and inhibits adipocyte differentiation and intracellular lipid accumulation in 3T3-L1 cells. PEE inhibits adipocyte differentiation by downregulating the expression of adipogenesis-related transcription factors via the MAPK pathways. Hence, PEE can be a potential natural product to treat obesity-related disorders.

## Figures and Tables

**Table 1:** Total phenolic content, radical scavenging activity and reducing power of PEE.

	TPC^2)^(mg GAE/g)	DPPH radical scavenging activity (%, μg/ml)				ABTS radical scavenging activity (%, μg/ml)				Reducing power (absorbance value^4)^)			
		
		50	100	200	400	50	100	200	400	50	100	200	400
PEE^1)^	92.39±1.30^3)^	24.95±1.98^d^	46.14±1.25^c^	70.47±2.45^b^	79.37±0.76^a^	53.83±0.39^c^	86.38±0.39^b^	>99.99^a^	>99.99^a^	0.081±0.01^d^	0.121±0.00^c^	0.183±0.01^b^	0.276±0.02^a^
BHA^5)^		54.98±2.38^c^	77.81±1.67^b^	86.14±0.78^a^	88.40±0.29^a^	>99.99^a^	>99.99^a^	>99.99^a^	>99.99^a^	0.325±0.00^d^	0.486±0.01^c^	0.685±0.02^b^	0.760±0.01^a^
AA^5)^		86.27±0.19^d^	91.29±0.22^c^	92.16±0.11^b^	93.73±0.29^a^	>99.99^a^	>99.99^a^	>99.99^a^	>99.99^a^	0.262±0.01^d^	0.532±0.01^c^	0.646±0.02^b^	0.773±0.01^a^

^1)^PEE: *Polygonum cuspidatum* 50% ethanol extract.

^2)^TPC: total phenolic content was expressed in terms of mg gallic acid equivalents.

^3)^Data represent the means ± SD in triplicate.

^4)^Absorbance value: absorbance value at 700 nm.

^5)^Positive controls used were butylated hydroxy anisole (BHA) and ascorbic acid (AA), respectively.

^a-d^: Means with the different superscripts within the same row are significantly different at *p* < 0.05.

**Table 2:** List of primers used for quantitative real-time PCR analysis.

Primers	Sequences (5’ → 3’)
C/EBPα	
Forward	CCTTCAACGACGAGTTCCTG
Reverse	TGGCCTTCTCCTGCTGCT
PPARγ	
Forward	TCGCTGATGCACTGCCTATG
Reverse	GACAGGTCCACAGAGCTGATT
SREBP-1c	
Forward	GGAGACATCGCAAACAAGCTC
Reverse	CAGACTGCAGGCCAGATCCA
ACC	
Forward	GACTGACTGCCGAAACATCTCTG
Reverse	GCCTCTTCCTGACAAACGAGT
FAS	
Forward	ATCCTGGAACGAGAACACGATCT
Reverse	AGAGACGTGTCACTCCTGGACCT
aP2	
Forward	AACACCGAGATTTCCTTCAA
Reverse	TCACGCCTTTCATAACACAT
β-actin	
Foward	GTTGGACCTGACAGACTACCTCA
Reverse	GTTGCCAATAGTGATGACCT
